# Depression Risk in Type 1 Versus Type 2 Diabetes: Cross‐Sectional Analysis of Body Mass Index (BMI) in a Nationally Diverse Cohort

**DOI:** 10.1002/edm2.70172

**Published:** 2026-02-11

**Authors:** Natalia Cruz‐Vespa, Michael L. Thomas, Michael J. McCarthy, Alejandro D. Meruelo

**Affiliations:** ^1^ Department of Psychology Wesleyan University Middletown Connecticut USA; ^2^ Department of Psychology Colorado State University Fort Collins Colorado USA; ^3^ Department of Psychiatry University of California San Diego, VA San Diego Healthcare System San Diego California USA; ^4^ Department of Psychiatry University of California San Diego La Jolla California USA

**Keywords:** body mass index, depression, type 1 diabetes, type 2 diabetes

## Abstract

**Introduction:**

Major depressive disorder (MDD) commonly co‐occurs with diabetes, but comparative risk across type 1 diabetes (DM1), type 2 diabetes (DM2) and non‐diabetic groups—and the role of body mass index (BMI)—remains uncertain.

**Methods:**

Using All of Us Research Program data, adults were classified as DM1, DM2 or non‐diabetic. Multivariable logistic regression estimated odds of MDD adjusting for age, sex at birth, race and ethnicity; BMI was added in secondary models. Effect modification by sex and race was tested. Structural equation modelling (SEM) assessed whether BMI statistically explained group differences.

**Results:**

In models excluding BMI, both DM1 and non‐diabetic participants had higher odds of MDD than DM2 (DM1 vs. DM2: OR = 1.53, 95% CI 1.17–1.99; non‐diabetic vs. DM2: OR = 1.20, 95% CI 1.16–1.25). Interactions by sex and race were significant; contrasts were stronger among females and heterogeneous across race strata. Adding BMI yielded directionally consistent group estimates and confirmed an independent association of higher BMI with higher MDD odds. SEM indicated statistical suppression for the non‐diabetic vs. DM2 contrast: non‐diabetic status related to lower BMI, while higher BMI related to higher MDD, producing a small indirect effect (~8%). The indirect path for DM1 vs. DM2 was non‐significant.

**Conclusions:**

Compared with DM2, both DM1 and non‐diabetic groups show higher adjusted odds of MDD. BMI is independently related to MDD but only modestly—and partly suppressively—accounts for the non‐diabetic vs. DM2 contrast. Findings support subgroup‐aware screening and the need for longitudinal data to clarify mechanisms.

## Graphical abstract

In a national All of Us cohort, major depressive disorder was more common in type 1 diabetes and non‐diabetic participants than type 2 diabetes. BMI was independently associated with depression and modestly suppressed non‐diabetic versus type 2 diabetes differences.

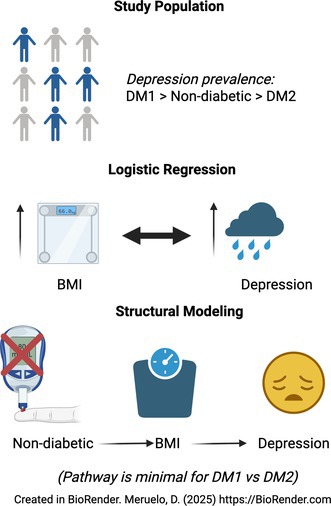

## 1 Introduction

Major depressive disorder (MDD) is one of the most common psychiatric conditions and is frequently associated with chronic physical illnesses, including diabetes mellitus [1, 2, 3, 4]. The co‐occurrence of depression and diabetes is linked to poorer glycemic control, reduced treatment adherence, increased risk of complications and greater healthcare utilisation [1, 5, 6, 7]. Although substantial research has examined the relationship between type 2 diabetes (DM2) and depression [8], comparatively less attention has been paid to MDD among individuals with type 1 diabetes (DM1)—despite growing recognition that DM1 involves unique psychological and physiological challenges. Prior studies of MDD in people with DM1 have often relied on small, clinically recruited or demographically narrow samples, limiting generalisability.

Existing literature suggests that individuals with DM1 may experience elevated rates of depression relative to the general population [9, 10, 11, 12, 13]. However, direct comparisons between DM1 and DM2 remain inconclusive, in part due to fundamental differences in disease aetiology and management. DM1 is an autoimmune condition typically diagnosed in childhood or adolescence and involves lifelong insulin therapy and ongoing self‐management responsibilities. By contrast, DM2 usually emerges in adulthood and is characterised by insulin resistance and metabolic dysfunction [14]. These distinctions suggest that the correlates of depression may differ between diabetes types, with psychosocial and self‐management demands possibly contributing more to MDD risk in individuals with DM1 [12, 13], and metabolic factors, such as adiposity and inflammation [15, 16, 17, 18], contributing more prominently in DM2.

Body mass index (BMI), a key marker of adiposity, is associated with both DM2 and MDD [17, 18]. However, it is unclear whether BMI partially explains differences in MDD prevalence across individuals with DM1, DM2 and non‐diabetic participants. Exploring BMI as a variable indirectly associated with MDD across diabetes groups may clarify whether distinct patterns of association are present, and whether psychosocial versus metabolic factors differ by diabetes type [16, 17, 18]. Understanding these differences can help inform more tailored mental health screening and support strategies for people with diabetes.

To address these questions, we conducted a cross‐sectional analysis using electronic health record data from the All of Us Research Program [19], a large, nationally diverse cohort. We compared MDD prevalence and adjusted odds among individuals with DM1, DM2 and non‐diabetic participants, and examined whether BMI could partially explain group differences. We anticipated heterogeneity by sex and race/ethnicity and expected BMI to be more closely tied to contrasts involving DM2 given its metabolic profile. By analysing DM1 and DM2 within a unified framework, we aimed to describe shared and distinct patterns linking diabetes status, adiposity and depression across demographic strata. Because depression and diabetes burdens differ across racial and ethnic groups, and minority populations often face barriers to mental‐health care [20, 21, 22], contextualising findings across demographic strata remains essential.

## 2 Methods

### 2.1 Data Source (Table 1)

**TABLE 1:** Participant characteristics—analytic sample.

Characteristic	DM2 (*n* = 62,123)	Control (*n* = 410,908)	DM1 (*n* = 730)	*p*	SMD
Age, mean (SD)	65.40 (12.72)	55.60 (16.95)	51.22 (17.91)	**< 0.001**	0.606
Sex at birth, *n* (%)					
Female	35,189 (56.6)	252,532 (61.5)	434 (59.5)	**< 0.001**	0.071
Male	26,123 (42.1)	154,228 (37.5)	288 (39.5)		
No matching concept	49 (0.1)	184 (0.0)	—		
Not male, not female, prefer not to answer or skipped	762 (1.2)	3964 (1.0)	—		
Race, *n* (%)					
Another single population	1605 (2.6)	8081 (2.0)	—	**< 0.001**	0.312
Asian	1256 (2.0)	14,800 (3.6)	—		
Black or African American	14,980 (24.1)	68,563 (16.7)	89 (12.2)		
I prefer not to answer	374 (0.6)	2214 (0.5)	—		
More than one population	2847 (4.6)	18,305 (4.5)	38 (5.2)		
None Indicated	11,407 (18.4)	63,140 (15.4)	85 (11.6)		
None of these	686 (1.1)	4090 (1.0)	—		
PMI: Skip	876 (1.4)	4778 (1.2)	—		
White	28,092 (45.2)	226,937 (55.2)	472 (64.7)		
Ethnicity, *n* (%)					
Hispanic or Latino	12,969 (20.9)	76,424 (18.6)	112 (15.3)	**< 0.001**	0.126
No matching concept	—	—	—		
Not Hispanic or Latino	47,218 (76.0)	323,399 (78.7)	604 (82.7)		
PMI: prefer not to answer	374 (0.6)	2214 (0.5)	—		
PMI: skip	876 (1.4)	4778 (1.2)	—		
Race/Ethnicity none of these	686 (1.1)	4090 (1.0)	—		
BMI, mean (SD)	33.78 (8.27)	29.21 (7.35)	28.92 (6.75)	**< 0.001**	0.423
MDD diagnosis, *n* (%)					
No	58,981 (94.9)	386,601 (94.1)	671 (91.9)	**< 0.001**	0.082
Yes	3142 (5.1)	24,307 (5.9)	59 (8.1)		

*Note:* This table summarises demographics and covariates for the complete‐case analytic sample used in multivariable models (i.e., non‐missing age, sex at birth, race, ethnicity, BMI and MDD status). For context, full cohort sizes prior to exclusions were: DM1 = 808, DM2 = 67,640 and non‐diabetic (Control) = 431,810; the counts shown here reflect complete cases (e.g., DM1 = 730) after applying model‐availability criteria. Per All of Us disclosure policy, any cell with a count ≤ 20 is masked with ‘—’; column totals may not sum exactly due to suppression and rounding. Continuous variables are reported as mean (SD); categorical variables as *n* (%). *p*‐values are from ANOVA (continuous) or chi‐squared tests (categorical). Bolded values indicate *p* < 0.05. Standardised mean differences (SMDs) compare each group with DM2; |SMD| ≥ 0.10 indicates meaningful imbalance.

We conducted a cross‐sectional analysis using data from the All of Us Research Program Registered Tier Dataset v8 [19]. This nationally diverse dataset includes electronic health records (EHR), survey responses and demographic data from a diverse cohort of U.S. participants, collected between 2018 and 2025. Analyses were conducted in a secure cloud‐based environment using R (version 4.2.2) [23].

### 2.2 Cohort Definition

**FIGURE 1:**
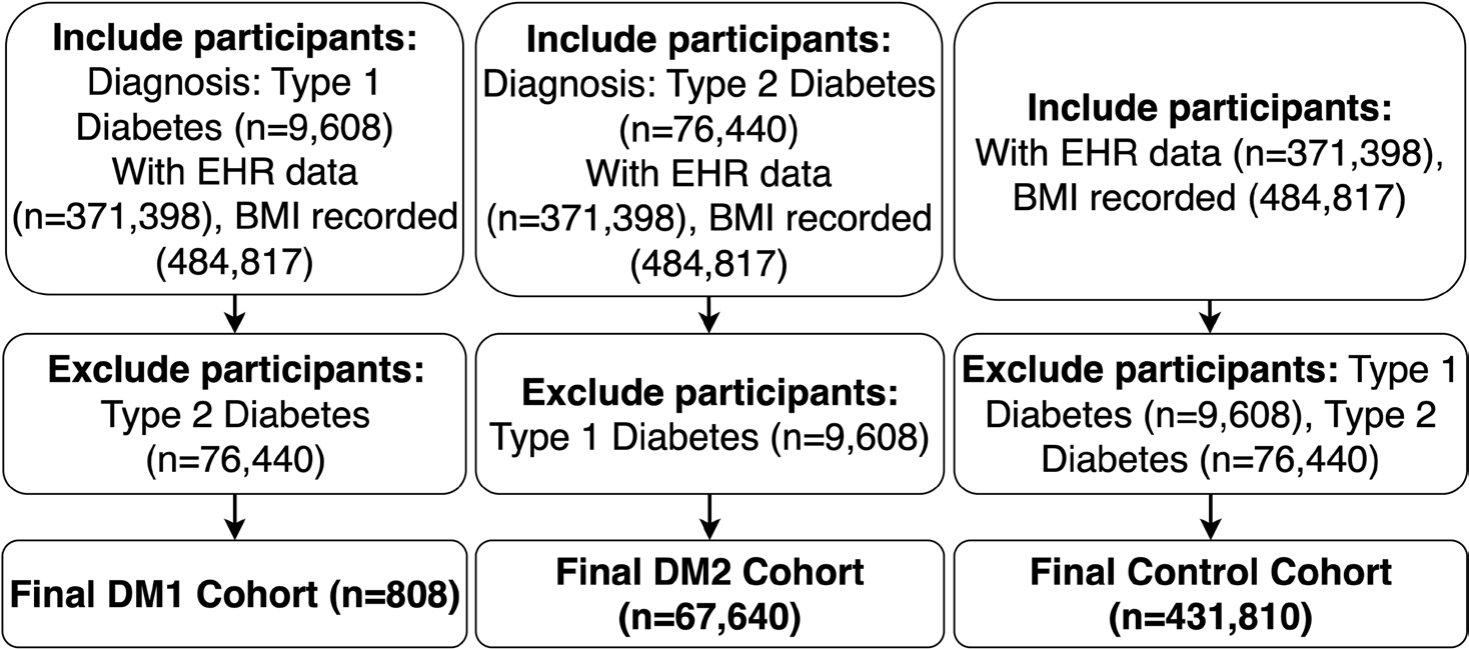
Participant inclusion and exclusion flowchart. Construction of analytic cohorts in the All of Us Researcher Workbench. Participants were identified using the Cohort Builder tool with parallel inclusion logic for each group. Type 1 Diabetes and Type 2 Diabetes cohorts were mutually exclusive, with cross‐exclusion of the alternate diabetes type. The control cohort included participants of all ages without diabetes, excluding any Type 1 or Type 2 Diabetes diagnoses. Final counts were 808 (Type 1 DM), 67,640 (Type 2 DM) and 431,810 (Controls), yielding a total analytic registry sample of 500,258.

We defined three groups: individuals with type 1 diabetes (DM1), individuals with type 2 diabetes (DM2) and a non‐diabetic comparison group. DM1 and DM2 were identified using curated concept sets based on high‐confidence (‘rank‐1’) structured diagnostic codes from the condition occurrence table in the EHR. Participants were excluded if they had conflicting diabetes diagnoses or simultaneous evidence of both DM1 and DM2. The non‐diabetic group consisted of individuals without any diabetes diagnosis and with complete demographic and EHR data (Figure 1). While some prior work uses age‐ and sex‐matched designs, we prioritised maintaining the population‐level diversity of the All of Us sample; matching would have excluded a substantial number of eligible participants, particularly those with DM1.

To enable meaningful comparisons across diabetes types, all three groups were included in a unified analytic framework. This approach allowed us to evaluate whether BMI was indirectly associated with group differences in MDD diagnosis without assuming shared underlying mechanisms.

### 2.3 Outcome Variable

The primary outcome was the presence of major depressive disorder (MDD), identified using a curated SNOMED concept set comprising: 45,557,367 (single episode), 45,557,370 (recurrent), 440,383 (unspecified) and 318,877 (depressive disorder, major). Participants were coded as positive for MDD if at least one of these codes appeared in their condition occurrence record.

### 2.4 Covariates

Covariates included age (continuous), sex at birth, race and ethnicity (categorical, including ‘prefer not to answer’ and ‘PMI: skip’ responses). BMI was modelled as a continuous variable, drawn from the most recent available measurement using concept ID 3038553. Variables were harmonised across groups. BMI was analysed as a variable potentially associated with group differences in MDD but not assumed to be causally ordered in time due to the cross‐sectional nature of the dataset.

In sensitivity analyses, we additionally considered glycosylated haemoglobin (HbA1c) as a proxy for diabetes severity. HbA1c values were drawn from the OMOP Measurement domain, restricted to % units and plausible clinical ranges (3%–20%). For participants with multiple values, we used the most recent value (any time); in a secondary specification we required HbA1c to fall within ±365 days of the BMI assessment. We also examined clinical categories (< 5.7%, 5.7%–6.4%, ≥ 6.5%).

### 2.5 Statistical Analysis

#### 2.5.1 Logistic Regression Models

We estimated odds of MDD across diabetes groups using logistic regression [24], with DM2 as the reference category. All models adjusted for age, sex at birth, race and ethnicity; a secondary model additionally included BMI to assess whether adiposity explained observed associations.

Categorical variables were entered as factors in R using default treatment coding. Reference categories were DM2 (diabetes group), female (sex at birth), White (race) and non‐Hispanic (ethnicity). We selected DM2 as the reference group because it is the largest and most clinically stable diabetes cohort in this dataset, providing the most precise baseline for contrasts; it is also the usual clinical comparator for DM1 and a relevant benchmark for non‐diabetic participants receiving cardiometabolic care [25].

Odds ratios (ORs) and 95% confidence intervals (CIs) were derived from model coefficients [26]. Statistical significance was assessed using two‐sided Wald tests, with *p* < 0.05 considered significant [27].

### 2.6 Sensitivity: HbA1c Adjustment

To evaluate whether proxies of glycemic severity altered group associations, we reestimated the primary logistic models with HbA1c included as an additional covariate under three prespecified specifications: (i) HbA1c as a continuous predictor using the latest available measurement (‘any‐time’), (ii) HbA1c as a continuous predictor restricted to measurements obtained within ±365 days of the BMI assessment, and (iii) HbA1c modelled categorically (< 5.7%, 5.7%–6.4%, ≥ 6.5%). These models used complete cases for all variables included in each specification (i.e., the analysis set shrank when an HbA1c value was required). Because HbA1c may lie on the causal pathway between diabetes group and MDD and the design is cross‐sectional, these are interpreted as sensitivity analyses to assess robustness rather than causal adjustment for disease severity. Odds ratios and 95% CIs were obtained from model coefficients using two‐sided Wald tests (*α* = 0.05).

### 2.7 Effect‐Modification Analyses

We assessed effect modification by sex at birth and race using likelihood‐ratio tests [28] comparing models with and without the interaction terms (group × sex; group × race). Given evidence of heterogeneity, we fit stratified logistic regression models with DM2 as the reference. In sex‐stratified models (Female and Male strata), we adjusted for age, race, ethnicity and BMI. In race‐stratified models, we adjusted for age, sex at birth, ethnicity and BMI. To improve stability for race strata, we collapsed race a priori to White, Black or African American, Asian, More than one population and Other/Unknown (the latter treated as exploratory due to mixed/missing coding). Odds ratios (ORs) and 95% confidence intervals (CIs) were reported from two‐sided Wald tests with *α* = 0.05.

### 2.8 Structural Equation Models (SEM)

We used structural equation modelling to test whether BMI was indirectly associated with group differences in MDD [29]. Two group contrasts were examined: (1) DM2 vs. non‐diabetic participants and (2) DM2 vs. DM1. The SEM framework specified one path from diabetes group to BMI and another from BMI to MDD, adjusting for all covariates listed above. Group coding was directional: for example, a negative coefficient from group to BMI indicated that the reference group (DM2) had higher BMI. Consistent with the regression models, DM2 served as the reference category for group coding.

Direct, indirect and total effects were estimated using the lavaan package [29] with robust maximum likelihood estimation (MLR) [30]. The proportion of the total effect indirectly associated with BMI was calculated as the ratio of the indirect to total effect. Given the cross‐sectional nature of the data, these analyses should be interpreted as estimates of statistical association rather than mediation in a causal or temporal sense [31].

### 2.9 Missing Data Handling

Participants with missing values for age, group, sex at birth, race, ethnicity or BMI were excluded using casewise deletion. Participants with implausible or conflicting diabetes diagnoses were also excluded. Mediation models were fit using complete case data for all included variables. HbA1c was not imputed; HbA1c‐adjusted models were fit on complete cases for HbA1c, leading to smaller analysis sets for those specifications.

### 2.10 Matched Sensitivity Analysis (Control vs. DM2)

As a robustness check for the Control vs. DM2 contrast, we performed 1:1 nearest‐neighbour propensity‐score matching with the ATT estimand [32]. The propensity score included age and sex at birth; we enforced exact matching on sex and 5‐year age bins, used a 0.05 calliper on the logit of the propensity score, and disallowed replacement. This analysis was limited to participants with sex at birth recorded as Female or Male to enable exact matching. Covariate balance was evaluated using standardised mean differences (SMDs), considering SMD < 0.10 acceptable. Outcomes were analysed using conditional logistic regression stratified by matched pair and adjusting for BMI; two‐sided Wald tests at *α* = 0.05 were used to infer statistical significance.

### 2.11 Sensitivity Analysis Using Multiple Imputation

To evaluate the impact of missing BMI data, we performed a sensitivity analysis using multiple imputation by chained equations (MICE) [33]. A 20% stratified subsample (~47,000 participants) was drawn from the full analytic dataset, preserving diabetes group and BMI missingness patterns. BMI values were imputed using predictive mean matching (method = ‘pmm’) with five imputations (*m* = 5) and five iterations per imputation. The imputation model included all primary analysis variables: diabetes group, age, sex at birth, race, ethnicity and MDD status. Logistic models were fit within each imputed dataset and combined using Rubin's rules [34] via the pool() function in the mice package [33].

We selected MICE over full information maximum likelihood (FIML) [35] given evidence that BMI missingness varied by group, sex and race—violating the assumption of data missing completely at random. Because BMI served as a mediator, MICE enabled direct imputation of mediator values, offering greater flexibility than FIML. This approach allowed us to assess whether excluding individuals with missing BMI biassed our effect estimates.

### 2.12 Software and Reproducibility

All data preprocessing and modelling were conducted in R [23] using the dplyr, lubridate, stats [23] and lavaan [29] packages. Confidence intervals and effect estimates were computed using robust standard errors [36] where appropriate.

### 2.13 Ethics Approval and Patient Consent Statement

This research was based on data from the All of Us Research Program, for which all participants gave informed consent covering use of their electronic health records, survey data and biospecimens. All procedures adhered to the ethical standards set forth in the Declaration of Helsinki and were carried out within the secure All of Us Researcher Workbench. The programme operates under the oversight of a central Institutional Review Board (IRB), which reviewed and approved all data collection and access procedures.

## 3 Results

We identified 500,258 participants across three cohorts prior to any exclusions: DM1 (*n* = 808), DM2 (*n* = 67,640) and non‐diabetic controls (*n* = 431,810). Table 1 presents characteristics for the complete‐case analytic sample used in multivariable models (e.g., DM1 *n* = 730, DM2 *n* = 62,123, Control *n* = 410,908), while Figure 1 details the inclusion criteria and sequential exclusions from the full cohort to the analytic sample. In the full pre‐exclusion cohort, the crude prevalence of MDD was highest in DM1 (7.5%), intermediate in controls (6.0%) and lowest in DM2 (5.0%).

A multivariable logistic regression model (Table 2a) adjusting for age, sex at birth, race and ethnicity showed higher odds of MDD in both DM1 and non‐diabetic groups compared with DM2, which served as the reference group. Specifically, DM1 had greater odds than DM2 (OR = 1.53, 95% CI 1.17–1.99, *p* = 0.0017) and the non‐diabetic group also had higher odds than DM2 (OR = 1.20, 95% CI 1.16–1.25, *p* < 2 × 10^−16^).

**TABLE 2a:** Association between diabetes group and MDD (excluding BMI).

Variable	Estimate	Std. error	OR [95% CI]	*p*
Intercept	−3.224	0.060	0.04 [0.04, 0.04]	**< 0.001**
Group: DM1	0.423	0.135	1.53 [1.17, 1.99]	**0.0017**
Group: Non‐diabetic	0.186	0.019	1.20 [1.16, 1.25]	**< 0.001**
Age (per year)	0.003	0.000	1.00 [1.00, 1.00]	**< 0.001**
Sex at birth: Male	−0.462	0.013	0.63 [0.61, 0.65]	**< 0.001**
Sex: Not male/female/prefer not to answer/skipped	−0.146	0.061	0.86 [0.77, 0.97]	**0.017**
Sex: No matching concept	−0.032	0.265	0.97 [0.58, 1.63]	0.905
Race: Asian	−0.876	0.070	0.42 [0.36, 0.48]	**< 0.001**
Race: Black or African American	0.053	0.048	1.05 [0.96, 1.16]	0.270
Race: I prefer not to answer	0.427	0.094	1.53 [1.28, 1.84]	**< 0.001**
Race: More than one population	0.230	0.053	1.26 [1.13, 1.40]	**< 0.001**
Race: None Indicated	0.021	0.057	1.02 [0.91, 1.14]	0.713
Race: None of these	0.324	0.080	1.38 [1.18, 1.62]	**< 0.001**
Race: PMI: Skip	0.229	0.079	1.26 [1.08, 1.47]	**0.0035**
Race: White	0.274	0.047	1.32 [1.20, 1.44]	**< 0.001**
Ethnicity: No matching concept	1.890	1.178	6.62 [0.66, 66.59]	0.108
Ethnicity: Not Hispanic or Latino	0.125	0.036	1.13 [1.05, 1.22]	**0.00064**

*Note:* Adjusted odds ratios from a multivariable logistic regression predicting major depressive disorder (MDD) diagnosis across diabetes cohorts, excluding BMI. The model includes diabetes group (DM1, DM2 non‐diabetic), age, sex at birth, race and ethnicity. Reference categories are DM2 (diabetes group), female (sex at birth), White (race) and Hispanic (ethnicity). Odds ratios (ORs) and 95% confidence intervals (CIs) are exponentiated coefficients. Statistical significance was assessed using two‐sided Wald tests, with *p* < 0.05 considered significant; bolded values indicate *p* < 0.05. Ethnicity categories with insufficient data were excluded due to singularities.

Several demographic correlates of MDD were also observed in this primary model (Table 2a). Increasing age was associated with a very small increase in odds (OR ≈ 1.003 per year, *p* < 0.001). Male sex at birth had lower odds than female (OR = 0.63, 95% CI 0.61–0.65, *p* < 0.001). Relative to White participants, Asian participants had lower odds (OR = 0.42, 95% CI 0.36–0.48, *p* < 0.001), while several non‐White, non‐Asian categories showed higher odds—for example, ‘More than one population’ (OR = 1.26, 95% CI 1.13–1.40, *p* < 0.001), ‘None of these’ (OR = 1.38, 95% CI 1.18–1.62, *p* < 0.001) and ‘PMI: Skip’ (OR = 1.26, 95% CI 1.08–1.47, *p* = 0.0035); Black or African American participants did not differ significantly from White (OR = 1.05, 95% CI 0.96–1.16, *p* = 0.270). Participants who were not Hispanic or Latino had modestly higher odds than Hispanic/Latino (OR = 1.13, 95% CI 1.05–1.22, *p* = 0.00064).

In a secondary model that additionally included body mass index (BMI; Table 2b), higher BMI was associated with greater odds of MDD (OR = 1.011 per unit, 95% CI 1.009–1.013, *p* < 0.001). After adding BMI, both diabetes‐group contrasts were significant with DM2 as the reference: DM1 vs. DM2 (OR = 1.73, 95% CI 1.32–2.27, *p* = 6.8 × 10^−5^) and non‐diabetic vs. DM2 (OR = 1.24, 95% CI 1.19–1.29, *p* < 0.001). These estimates were obtained in a complete‐case analysis restricted to participants with observed BMI (*n* = 473,761).

**TABLE 2b:** Association between diabetes group and MDD (including BMI).

Variable	Estimate	Std. error	OR [95% CI]	*p*
Intercept	−3.673	0.070	0.025 [0.022, 0.029]	**< 0.001**
Group: DM1	0.548	0.138	1.730 [1.321, 2.266]	**6.84 × 10** ^**−5**^
Group: Non‐diabetic	0.212	0.020	1.236 [1.188, 1.287]	**1.60 × 10** ^**−25**^
Age (per year)	0.00359	0.000395	1.004 [1.003, 1.004]	**< 0.001**
Sex at birth: Male	−0.446	0.0138	0.640 [0.623, 0.658]	**< 0.001**
Sex: Not male/female/prefer not to answer/skipped	−0.117	0.0625	0.890 [0.787, 1.006]	0.061
Sex: No matching concept	0.0068	0.264	1.007 [0.600, 1.689]	0.979
Race: Asian	−0.796	0.0739	0.451 [0.390, 0.522]	**4.81 × 10** ^**−27**^
Race: Black or African American	0.076	0.0515	1.080 [0.976, 1.194]	0.137
Race: I prefer not to answer	0.480	0.0983	1.616 [1.333, 1.960]	**1.06 × 10** ^**−6**^
Race: More than one population	0.256	0.0565	1.292 [1.157, 1.443]	**5.67 × 10** ^**−6**^
Race: None indicated	0.065	0.0603	1.067 [0.948, 1.201]	0.282
Race: None of these	0.366	0.0842	1.442 [1.223, 1.701]	**1.37 × 10** ^**−5**^
Race: PMI: skip	0.294	0.0822	1.342 [1.142, 1.577]	**3.45 × 10** ^**−4**^
Race: White	0.313	0.0498	1.368 [1.241, 1.508]	**3.04 × 10** ^**−10**^
Ethnicity: No matching concept	2.416	1.245	11.203 [0.976, 128.638]	0.052
Ethnicity: Not Hispanic or Latino	0.138	0.0384	1.148 [1.065, 1.238]	**3.26 × 10** ^**−4**^
BMI (kg/m^2^)	0.0109	0.000798	1.011 [1.009, 1.013]	**< 0.001**

*Note:* Adjusted odds ratios from a multivariable logistic regression predicting major depressive disorder (MDD) diagnosis across diabetes cohorts, including BMI. The model adjusts for diabetes group (DM1, DM2 and non‐diabetic), age, sex at birth, race, ethnicity and BMI. Categorical variables were coded as factors using treatment coding. Reference groups are DM2 (diabetes group), female (sex at birth), White (race) and Hispanic (ethnicity). Odds ratios (ORs) and 95% confidence intervals (CIs) are derived from exponentiated coefficients. Statistical significance was assessed using two‐sided Wald tests, with *p* < 0.05 considered significant; bolded values indicate *p* < 0.05. Ethnicity categories with insufficient data were excluded due to singularities.

In sensitivity analyses additionally adjusting for HbA1c (a proxy for glycemic control), the Control vs. DM2 association strengthened relative to the BMI‐only model (BMI only: OR = 1.24, 95% CI 1.19–1.29, *p* = 1.60 × 10^−25^). Adding HbA1c as a continuous covariate using the most recent value (‘any time’) yielded OR = 2.01 (95% CI 1.91–2.13, *p* = 1.77 × 10^−137^; *n* = 130,178), with similar results when restricting HbA1c to measurements within ±365 days of the BMI assessment (OR = 1.91, 95% CI 1.72–2.12, *p* = 1.04 × 10^−34^; *n* = 42,087) and when modelling HbA1c in clinical categories (OR = 2.08, 95% CI 1.98–2.19, *p* = 4.93 × 10^−165^; *n* = 130,178). For DM1 vs. DM2, estimates were elevated and statistically significant when HbA1c was included as a continuous (OR = 1.62, 95% CI 1.14–2.31, *p* = 0.0077) or categorical covariate (OR = 1.62, 95% CI 1.13–2.31, *p* = 0.0082), but attenuated and imprecise under the ±365‐day restriction (OR = 0.56, 95% CI 0.21–1.51, *p* = 0.25). Because HbA1c may lie on the causal pathway, these are presented as sensitivity analyses; notably, the Control vs. DM2 contrast was robust—and larger—across all HbA1c specifications.

**FIGURE 2:**
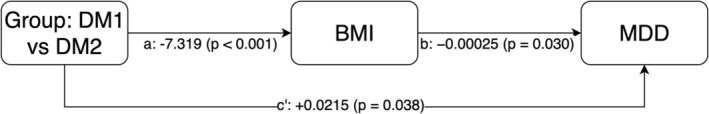
Mediation model testing whether BMI mediates the association between diabetes group (DM1 vs. DM2) and major depressive disorder (MDD). The indirect effect (*a* × *b*) was small but statistically detectable (+0.00184, *p* ≈ 0.031), accounting for ≈8% of the total effect. The BMI → MDD path was near zero in magnitude (*b* ≈ −0.00025), indicating only a modest statistical (inconsistent mediation/suppression) pattern rather than a clinically meaningful mediated pathway.

**FIGURE 3:**
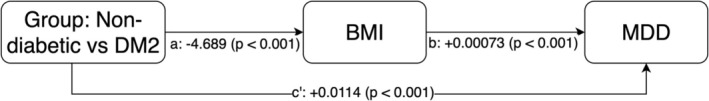
Mediation model testing whether BMI mediates the association between diabetes group (Non‐diabetic vs. DM2) and major depressive disorder (MDD). The path from group to BMI was negative and significant (*a* = −4.689, *p* < 0.001) and the path from BMI to MDD was positive and significant (*b* = +0.00073, *p* < 0.001), yielding a small but significant negative indirect effect (*a* × *b* = −0.00340, *p* < 0.001). The direct effect remained positive and significant (*c*′ = +0.0114, *p* < 0.001), with an overall total effect of +0.0080. Thus, BMI acted as a suppressor, offsetting ~43% of the total association (−0.00340/0.0080), that is, statistical suppression rather than explanatory mediation.

To evaluate whether BMI statistically mediated cohort differences in MDD (Table 3; Figures 2 and 3), we fit structural equation models after multiple imputation of BMI. For the Control vs. DM2 contrast, the group → BMI path was strongly negative (*β* = −4.69, *p* < 0.001) and the BMI → MDD path was positive (*β* = 7.26 × 10^−4^, *p* < 0.001), yielding a negative indirect effect (a × b = −0.00340, *p* < 0.001) and a positive total effect (0.00799, *p* < 0.001); thus BMI operated as a suppressor, offsetting ~43% of the total association. For DM1 vs. DM2, the group → BMI path was also negative (*β* = −7.32, *p* < 0.001) while BMI → MDD was small and negative (*β* = −2.51 × 10^−4^, *p* = 0.030), producing a modest positive indirect effect (0.00184, *p* = 0.030) and a positive total effect (0.0234, *p* = 0.023), corresponding to ~7.9% mediated.

**TABLE 3:** Mediation of diabetes group differences in MDD via BMI (SEM results).

Comparison	*a*: Group → BMI	*b*: BMI → MDD	Indirect effect	Direct effect (*c*′)	Total effect	‘Proportion mediated’^a^
Non‐diabetic vs. DM2	−4.689 **(*p* < 0.001)**	+0.000726 **(*p* < 0.001)**	−0.00340 **(*p* < 0.001)**	+0.01139 **(*p* < 0.001)**	+0.00799 **(*p* < 0.001)**	−42.6% (suppression)
DM1 vs. DM2	−7.319 **(*p* < 0.001)**	−0.000251 **(*p* = 0.030)**	+0.00184 **(*p* = 0.031)**	+0.0215 **(*p* = 0.038)**	+0.0234 **(*p* = 0.023)**	7.9%

*Note:* Structural equation models (SEM) estimating indirect (*a* × *b*), direct (*c*′), and total effects of diabetes group on MDD, with BMI as the proposed mediator. Models adjust for age, sex at birth, race and ethnicity. Coding: non‐diabetic vs. DM2 uses 0 = DM2, 1 = non‐diabetic; DM1 vs. DM2 uses 0 = DM2, 1 = DM1.

^a^ ‘Proportion mediated’ is computed as (indirect ÷ total). Negative values indicate statistical suppression (the BMI pathway operates in the opposite direction of the total effect, offsetting part of the association), not explanatory mediation. Bold values denote *p* < 0.05.

In effect‐modification analyses, the cohort–MDD association varied by sex and race. Likelihood‐ratio tests supported both interactions (group × sex: ΔDeviance = 1094.6 on 9 df, *p* < 2 × 10^−16^; group × race: ΔDeviance = 794.8 on 12 df, *p* < 2 × 10^−16^). In sex‐stratified models using DM2 as the reference, females showed higher odds of MDD for both contrasts—Control vs. DM2 (OR = 1.23, 95% CI 1.17–1.30, *p* = 3.17 × 10^−17^; BH‐adjusted *p* = 1.27 × 10^−16^) and DM1 vs. DM2 (OR = 1.78, 95% CI 1.28–2.46, *p* = 5.33 × 10^−4^; BH = 7.11 × 10^−4^). Among males, Control vs. DM2 remained elevated (OR = 1.22, 95% CI 1.14–1.31, *p* = 2.46 × 10^−8^; BH = 4.91 × 10^−8^), whereas DM1 vs. DM2 did not meet the 0.05 threshold (OR = 1.58, 95% CI 0.96–2.59, *p* = 0.071).

Race‐stratified models (collapsed a priori for stability) also indicated heterogeneity: Asian participants showed lower odds for Control vs. DM2 (OR = 0.66, 95% CI 0.46–0.95, *p* = 0.023; BH = 0.044); Black or African American participants had substantially higher odds for DM1 vs. DM2 (OR = 3.38, 95% CI 1.82–6.25, *p* = 1.07 × 10^−4^; BH = 2.68 × 10^−4^) and a smaller elevation for Control vs. DM2 (OR = 1.19, 95% CI 1.10–1.30, *p* = 5.47 × 10^−5^; BH = 1.82 × 10^−4^); participants reporting More than one population showed higher odds for Control vs. DM2 (OR = 1.22, 95% CI 1.02–1.46, *p* = 0.026; BH = 0.044); and among White participants, Control vs. DM2 was higher (OR = 1.38, 95% CI 1.31–1.46, *p* = 6.77 × 10^−29^) while DM1 vs. DM2 was near null (OR = 1.10, 95% CI 0.73–1.65, *p* = 0.649). Results for “Other/Unknown” race were noted as exploratory given mixed/missing coding, though DM1 vs. DM2 showed a large elevation (OR = 4.46, 95% CI 2.70–7.37, *p* = 5.39 × 10^−9^; BH = 2.70 × 10^−8^).

As a conservative robustness check for the Control vs. DM2 contrast, we performed 1:1 nearest‐neighbour propensity‐score matching with exact matching on sex and 5‐year age bins and a calliper of 0.05 (ATT). Post‐match balance was excellent (e.g., |SMD| for propensity‐score distance ≈ 0.045; for age ≈0.041). Conditional logistic regression within matched pairs (adjusting for BMI) showed a small but statistically significant elevation in Controls vs. DM2 (OR = 1.28, 95% CI 1.21–1.35, *p* = 4.30 × 10^−20^), supporting the robustness of the primary findings.

Sensitivity analyses using multiple imputation to address missing BMI data (Table S1) yielded consistent estimates with the complete‐case results, supporting the robustness of the primary findings.

## 4 Discussion

This study adds nuance to the complex—and sometimes conflicting—literature on depression in diabetes. In unadjusted comparisons, MDD prevalence was highest in DM1, followed by non‐diabetic participants and DM2. In adjusted models controlling for age, sex at birth, race and ethnicity, both DM1 and non‐diabetic groups had higher odds of MDD than DM2, with the DM1 vs. DM2 contrast more pronounced than the non‐diabetic vs. DM2 contrast. Thus, our findings align with prior reports of higher depression in DM1 relative to DM2—often attributed to earlier onset and intensive self‐management demands—while also identifying an elevated risk among non‐diabetic participants relative to DM2 [9, 37, 38, 39].

Differences between our estimates and some prior studies likely reflect population‐based sampling, EHR‐based case definitions, and unmeasured clinical or psychosocial factors (e.g., glycemic control, disease duration, healthcare access) that vary across cohorts [40, 41, 42, 43].

Two patterns help contextualise the unexpected finding that non‐diabetic participants have higher adjusted odds of MDD than DM2. First, DM2 participants in All of Us are embedded in ongoing medical care, which may facilitate depression recognition and management, selective non‐participation of the most severely depressed, or differential coding of psychiatric conditions (i.e., surveillance and diagnostic practice effects).

Second, behavioural and comorbidity profiles may differ: DM2 participants had higher BMI on average, yet BMI only partially explained the non‐diabetic vs. DM2 difference, suggesting additional mechanisms (e.g., care engagement, cardiometabolic treatment effects, or socioeconomic factors) may contribute. This pattern is also consistent with statistical suppression: BMI differences run counter to the direction of the non‐diabetic vs. DM2 association with MDD, so controlling for BMI reveals a somewhat larger direct group contrast.

Effect‐modification analyses showed that associations varied by sex and race. Using DM2 as the reference, the Control vs. DM2 contrast was elevated in both females and males, whereas the DM1 vs. DM2 contrast reached significance only in females. Across race strata (with prespecified collapsed categories), Control vs. DM2 was lower among Asian participants, and substantially higher DM1 vs. DM2 odds were observed among Black or African American participants, whereas Control vs. DM2 was higher among White participants. These findings indicate that pooled estimates can obscure subgroup patterns with potential clinical relevance.

Consistent with prior literature, Black or African American participants exhibited higher adjusted odds of MDD, whereas Asian participants had lower odds relative to White participants [20, 44, 45, 46]. These patterns likely reflect intersecting structural inequities, care access and cultural factors that influence diagnosis, stigma and treatment engagement, reinforcing the need for culturally responsive screening and follow‐up.

Adding BMI to the regression model showed that higher BMI was independently associated with greater MDD odds, aligning with links between adiposity, metabolic dysfunction, inflammation and depression [17, 18, 47]. Structural equation models suggested BMI statistically accounted for a modest portion of the non‐diabetic vs. DM2 difference in MDD (indirect effect small; ~8% of the total effect), while the direct effect remained significant. Because the non‐diabetic group had lower BMI than DM2 while higher BMI was associated with higher MDD, the indirect path operated opposite the direct path—consistent with statistical suppression—so adjusting for BMI tends to accentuate, not attenuate, the non‐diabetic vs. DM2 association. For DM1 vs. DM2, the indirect path through BMI was near zero and the direct effect was small but positive.

Thus, BMI differences alone are unlikely to be the primary driver of group contrasts; rather, BMI appears to play a limited statistical role for the non‐diabetic vs. DM2 comparison and little role for DM1 vs. DM2. Given the small magnitude of the indirect effect, its clinical significance is likely limited.

In sensitivity models additionally adjusting for HbA1c (a proxy for current glycemic control), the direction of the primary findings did not change, and the Control vs. DM2 contrast strengthened relative to BMI‐only models (e.g., OR ≈ 2.01 with any‐time HbA1c; OR ≈ 2.08 with clinical categories). For DM1 vs. DM2, estimates were elevated with any‐time and categorical HbA1c but attenuated and imprecise when restricting HbA1c to ±365 days of BMI owing to reduced sample size. Because HbA1c and MDD were measured cross‐sectionally, we interpret these as robustness checks rather than causal adjustments for disease severity.

As a further robustness check for potential confounding by age and sex, 1:1 nearest‐neighbour matching (exact on sex and 5‐year age bins; calliper 0.05) yielded good post‐match balance and a persistent elevation for Controls vs. DM2 (conditional OR = 1.28, 95% CI 1.21–1.35), consistent with the unmatched analyses. Together with the HbA1c sensitivity models, these results support the stability of the main findings.

Several limitations warrant emphasis. Given the cross‐sectional design, these paths should not be interpreted as causal mediation; rather, they reflect statistical associations—‘partially explained by’ BMI in general and, for the non‐diabetic vs. DM2 comparison, a statistical suppression pattern whereby BMI operates opposite the direct association. Directionality may also be bidirectional (e.g., depression leading to weight change and metabolic dysregulation).

Second, the DM1 subgroup was relatively small (*n* = 808), which limits power for DM1‐focused contrasts and increases uncertainty, particularly in stratified and time‐restricted sensitivity analyses.

Third, EHR diagnostic accuracy may differ for DM1 vs. DM2, with potential misclassification from code use, transitions in therapy, or clinician documentation patterns; this could attenuate or distort group contrasts.

Fourth, residual confounding remains possible given incomplete information on socioeconomic status, disease duration, psychiatric history and treatment exposures [40, 41, 42, 43].

Fifth, HbA1c values were ascertained at variable times relative to BMI and MDD coding and were not imputed, so HbA1c‐adjusted models used smaller, potentially selected subsamples; moreover, adjusting for HbA1c could over‐adjust or introduce collider bias if glycemic control is influenced by both diabetes type and depression.

In conclusion, in this large EHR‐based sample, both DM1 and non‐diabetic participants had higher odds of MDD than DM2 after adjustment for demographics, with the DM1–DM2 contrast larger than the non‐diabetic–DM2 contrast. BMI was independently associated with MDD and partially—but modestly—explained the non‐diabetic vs. DM2 difference. Subgroup analyses showed meaningful heterogeneity by sex and race.

These findings underscore the need to consider care engagement, diagnostic practices and demographic context when comparing depression across diabetes types. Prospective studies—with validated DM1/DM2 phenotyping, disease duration, richer psychosocial measures and repeated assessments of mood and glycemia—are needed to clarify mechanisms and inform targeted screening and treatment.

## Author Contributions

N.C.‐V. contributed to writing – review and editing. M.L.T. contributed to methodology and writing – review and editing. M.J.M. contributed to conceptualisation and writing – review and editing. A.D.M. contributed to conceptualisation, formal analysis, visualisation, writing – original draft, writing – review and editing and funding acquisition. All authors reviewed and approved the final manuscript.

## Funding

This research was funded by the National Institute on Alcohol Abuse and Alcoholism (NIAAA) under grant K23 AA026869, awarded to Alejandro D. Meruelo. Additional support was provided by the U.S. Department of Veterans Affairs through a VA Merit Award (BX003431) granted to Michael J. McCarthy.

## Conflicts of Interest

The authors declare no conflicts of interest.

## Supporting information


**Table S1:** Comparison of participants with complete vs. incomplete data.

## Data Availability

This research utilised the Registered Tier Dataset (version 8) from the All of Us Research Program. These data are accessible through the All of Us Researcher Workbench (https://www.researchallofus.org) to approved users. Due to participant privacy protections, the dataset is not publicly available; however, qualified researchers may gain access by completing the necessary training and complying with the All of Us Data and Statistics Dissemination Policy.
