# Supplementary material for: Depression Risk in Type 1 Versus Type 2 Diabetes: Cross‐Sectional Analysis of Body Mass Index (BMI) in a Nationally Diverse Cohort
Source: Endocrinol Diabetes Metab. 2026 Feb 11;9(2):e70172. doi: 10.1002/edm2.70172 (PMC12894052; doi:10.1002/edm2.70172)
Supplement: Supplementary file 1 — Table S1: Comparison of participants with complete vs. incomplete data. [file EDM2-9-e70172-s001.docx]

**Sensitivity Analysis Findings**

BMI was missing for ~5.3% of participants (26,497/500,258). Comparing complete versus incomplete cases showed meaningful composition differences (SMDs >0.10) for diabetes group (SMD = 0.209), sex at birth (SMD = 0.139), and race (SMD = 0.351), indicating potential selection bias from complete‐case analysis (**Supplementary Table 1** below). To assess robustness, we conducted multiple imputation (predictive mean matching, m = 5) on a 20% stratified subsample. Pooled estimates were directionally consistent with the primary models and, if anything, slightly stronger: relative to DM2, the Control group had higher odds of MDD (OR = 1.32, 95% CI 1.16–1.51, p = 0.0016) and DM1 also had higher odds (OR = 2.16, 95% CI 1.25–3.76, p = 0.0062). BMI showed a small but statistically significant association with MDD (per‐unit OR = 1.01, 95% CI approximating 1.00–1.02; p = 0.0058). Other covariate patterns mirrored the main analysis (e.g., lower odds for males: OR = 0.66, 95% CI 0.62–0.71, p < 10⁻²⁰; lower odds for Asian race: OR = 0.44, 95% CI 0.32–0.62, p = 2.1×10⁻⁶). Overall, imputing missing BMI did not materially change inferences about group differences or covariate effects, reinforcing the robustness of the complete‐case findings.

**Supplementary Table 1. Comparison of Participants With Complete vs. Incomplete Data**

| **Characteristic** | **Complete Data** | **Missing Data** | **p** | **SMD** |
| --- | --- | --- | --- | --- |
| **n** | 473,761 | 26,497 |  |  |
| **Group, n (%)** |  |  | **<0.001** | 0.209 |
| DM2 | 62,123 (13.1) | 5,517 (20.8) |  |  |
| Control | 410,908 (86.7) | 20,902 (78.9) |  |  |
| DM1 | 730 (0.2) | 78 (0.3) |  |  |
| **Age, mean (SD)** | 56.88 (16.79) | 54.79 (17.49) | **<0.001** | 0.122 |
| **Sex at birth, n (%)** |  |  | **<0.001** | 0.139 |
| Female | 288,155 (60.8) | 17,855 (67.4) |  |  |
| Male | 180,639 (38.1) | 8,449 (31.9) |  |  |
| No matching concept | 234 (0.0) | — |  |  |
| Not male/female/prefer not/Skipped | 4,733 (1.0) | 191 (0.7) |  |  |
| **Race, n (%)** |  |  | **<0.001** | 0.351 |
| Another single population | 9,699 (2.0) | 944 (3.6) |  |  |
| Asian | 16,075 (3.4) | 739 (2.8) |  |  |
| Black or African American | 83,632 (17.7) | 1,813 (6.8) |  |  |
| I prefer not to answer | 2,591 (0.5) | 171 (0.6) |  |  |
| More than one population | 21,190 (4.5) | 1,539 (5.8) |  |  |
| None Indicated | 74,632 (15.8) | 4,267 (16.1) |  |  |
| None of these | 4,778 (1.0) | 316 (1.2) |  |  |
| PMI: Skip | 5,663 (1.2) | 308 (1.2) |  |  |
| White | 255,501 (53.9) | 16,400 (61.9) |  |  |
| **Ethnicity, n (%)** |  |  | **<0.001** | 0.047 |
| Hispanic or Latino | 89,505 (18.9) | 5,421 (20.5) |  |  |
| No matching concept | — | — |  |  |
| Not Hispanic or Latino | 371,221 (78.4) | 20,280 (76.5) |  |  |
| PMI: Prefer Not To Answer | 2,591 (0.5) | 171 (0.6) |  |  |
| PMI: Skip | 5,663 (1.2) | 308 (1.2) |  |  |
| Race/Ethnicity None of These | 4,778 (1.0) | 316 (1.2) |  |  |
| **BMI, mean (SD)** | 29.81 (7.64) | NA | NA | NA |
| **MDD diagnosis, n (%)** |  |  | **<0.001** | 0.056 |
| No (0) | 448,310 (94.6) | 24,720 (93.3) |  |  |
| Yes (1) | 25,451 (5.4) | 1,777 (6.7) |  |  |

This table compares demographic and clinical characteristics between participants with complete data (all model covariates including BMI available) and those with missing data (≥1 model covariate missing). Percentages are column-wise. For continuous variables, values are mean (SD) and p-values come from two-sample tests (Welch’s t where appropriate); for categorical variables, values are n (%) and p-values come from χ² tests. Standardized mean differences (SMDs) summarize imbalance between the Complete vs. Missing columns (|SMD| > 0.10 is typically considered meaningful).

Per All of Us disclosure policy, any cell with n ≤ 20 is masked with an em dash (—); as a result, row totals may not sum exactly, and percentages may not precisely reflect masked counts. “NA” indicates the metric is undefined by design (e.g., BMI in the Missing Data column).
